# The Elliott-Yafet theory of spin relaxation generalized for large spin-orbit coupling

**DOI:** 10.1038/srep22706

**Published:** 2016-03-04

**Authors:** Annamária Kiss, Lénard Szolnoki, Ferenc Simon

**Affiliations:** 1 Institute for Solid State Physics and Optics, Wigner Research Centre for Physics, Hungarian Academy of Sciences, PO Box 49, H-1525 Budapest, Hungary; 2 BME-MTA Exotic Quantum Phases Research Group, Budapest University of Technology and Economics, Budapest, Hungary; 3 Department of Physics, Budapest University of Technology and Economics and MTA-BME Lendület Spintronics Research Group (PROSPIN), PO Box 91, H-1521 Budapest, Hungary

## Abstract

We generalize the Elliott-Yafet (EY) theory of spin relaxation in metals with inversion symmetry for the case of large spin-orbit coupling (SOC). The EY theory treats the SOC to the lowest order but this approach breaks down for metals of heavy elements (such as e.g. caesium or gold), where the SOC energy is comparable to the relevant band-band separation energies. The generalized theory is presented for a four-band model system without band dispersion, where analytic formulae are attainable for arbitrary SOC for the relation between the momentum- and spin-relaxation rates. As an extended description, we also consider an empirical pseudopotential approximation where SOC is deduced from the band potential (apart from an empirical scaling constant) and the spin-relaxation rate can be obtained numerically. Both approaches recover the usual EY theory for weak SOC and give that the spin-relaxation rate approaches the momentum-relaxation rate in the limit of strong SOC. We argue that this limit is realized in gold by analyzing spin relaxation data. A calculation of the *g*-factor shows that the empirical Elliott-relation, which links the *g*-factor and spin-relaxation rate, is retained even for strong SOC.

The prospect of using electron spins as information carriers, (a field known as spintronics) renewed the interest in the fundamental desciption of spin relaxation in metals and semiconductors. Spintronics devices rely on the controlled creation and readout of a non-equilibrium net spin population. Spin relaxation in turn characterizes how rapidly the non-equilibrium spin population decays, knowledge and theoretical description of spin relaxation is therefore of central importance.

The first experiment in the field dates back to 1955 (ref. 1), which reported the first electron spin resonance in metals. The first proper theoretical description of spin relaxation in metals with inversion symmetry was provided by Elliott^2^, which was later generalized to lower temperatures and for various relaxation mechanisms by Yafet^3^. The Elliott-Yafet (EY) theory of spin relaxation is valid for metals and semiconductors (metals in the following) with i) inversion symmetry, ii) weak spin-orbit coupling (SOC), and iii) low quasi-particle scattering rate^4^,^5^. When the inversion symmetry is broken, the so-called D’yakonov-Perel’ (DP) mechanism describes the spin relaxation^6^. The EY theory is also relevant for small-gap semiconductors (e.g. InSb), where the breaking of the inversion symmetry is weak and the spin relaxation due to the EY mechanism overcomes that due to the DP^4^,^5^. The case of sizeable quasi-particle scattering was described before^7^,^8^ but strong SOC has not been considered yet.

The conventional EY theory exploits that in the presence of inversion symmetry, the spin-up and spin-down states remain degenerate as a result of time-reversal invariance (or Kramers’ theorem) until the later is broken by e.g. a magnetic field. The presence of a nearby band gives rise to an admixture of the spin-up/down states in the conduction band, while the energy degenerancy is retained. As a result, the EY desciption is a four-band theory. The admixed states read:


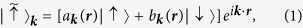



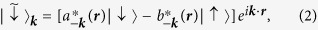


where |↑〉 and |↓〉 are the pure (unperturbed) spin states and 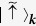, 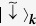 are the perturbed Bloch states. In the first order of the SOC, the coefficients are given by the *L* matrix element of the SOC for the conduction and the near lying band, and the corresponding energy separation Δ as: |*b*_***k***_|/|*a*_***k***_| ≈ *L*/Δ.

Elliott showed with a first-order time dependent perturbation treatment^2^ that the usual momentum scattering induces spin transitions for the admixed states, i.e. a spin relaxation. With Γ_s_ = *ħ*/*τ*_s_ and Γ = *ħ*/*τ* used for the spin- and momentum-relaxation rates (*τ*_s_ and *τ* are the corresponding relaxation times), respectively:


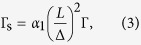


where *α*_1_ is a band structure dependent constant near unity.

Elliott further showed that the magnetic energy of the admixed states is different from that of the pure spin-states, i.e. there is a shift in the electron *g*-factor:


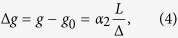


where 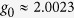 is the free electron *g*-factor, *α*_2_ is another band structure dependent constant near unity. Eqs (3) and (4) give the so-called Elliott relation


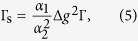


which links three empirical measurables; Γ_s_, Γ, and Δ*g*. In practice, the spin-relaxation time is obtained for metals from conduction electron spin resonance (CESR) measurements^1^ as: Γ_s_ = *ħγ*Δ*B*. Here ΔΒ is the homogeneous ESR line-width and *γ*/2*π* = 28.0 GHz/T is the electron gyromagnetic ratio. The CESR resonance line position yields the *g*-factor shift.

Monod and Beuneu tested empirically^9^ the validity of Eq. (3) and found that the atomic SOC induced energy splittings approximate well the appropriate matrix elements. For most elemental metals 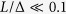 holds but for Cs, Cu, Ag, and In it is about 0.1 and for other metals 0.2 (Pb), 0.3 (Hg and Sn) with a 0.9 as the largest value in Au. Clearly, the validity of a first-order perturbation treatment of the SOC for these cases is questionable. This motivates us to revisit the EY theory for the case when the SOC is not small compared to other energy scales (kinetic energy or band separations).

Here, we discuss the most general form of the SOC Hamiltonian which is applicable for the EY model. We proceed with a simplified four-band Hamiltonian and solve the problem of spin relaxation in the first order of the scattering but exactly for the SOC. We find that the conventional EY result is recovered for weak SOC. A calculation of the *g*-factor yields the Elliott-relation for arbitrary strength of the SOC. A numerical calculation is also presented for the spin relaxation in the framework of a pseudopotential approximation for the conduction electrons, where SOC is obtained directly from the potential together with an empirical scaling factor. The numerical result qualitatively returns the result of the EY model calculations. The most important results are that the spin scattering time approaches the momentum scattering for strong SOC and that the Elliott relation remains valid irrespective of the SOC strength. We revisit previous experimental data on Au by Monod and Jánossy^10^ and show that these effects were observed already but it avoided the attention.

**The Elliott theory of spin relaxation**. We consider the Elliott mechanism of spin relaxation^2^ in which the conduction electron spin interacts with its motion in the electric field of the host lattice described by the periodic potential 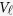. This lattice induced spin-orbit coupling, given by the Hamiltonian


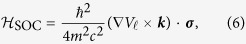


leads to the mixing of the originally pure spin states in the electron wave function. Thus, the conduction electron spins can now relax through ordinary momentum scattering caused by impurity atoms, for example. The spin-relaxation rate is estimated from the spin-flip transition probability 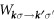 of an electron scattering on the impurity potential *V*, while the momentum-relaxation rate includes also the non spin-flip transition probability 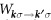. In this picture we neglect the SOC caused by the electric field of the impurity atoms, and also assume that they give rise to much larger momentum scattering than the host ions.

To formulate the above described Elliott mechanism, we start from the wave functions of electrons in the periodic potential *V*_ℓ_, which are Bloch-type as


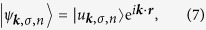


where *n* is the band index, and the Bloch functions 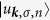 are lattice-periodic. Each band is at least two fold degenerate due to the presence of time-reversal symmetry.

The spin-flip transition probability 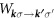 is determined by the matrix element 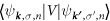 within a first-order perturbation theory, and in parallel, the non spin-flip transition element 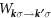 is related to the matrix element 
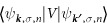
. If we assume that the impurity scattering potential *V* is slowly varying on the scale of the unit cell, the matrix elements can be approximated by the factorization


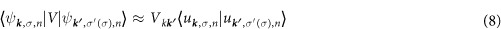


with 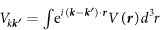, which gives the spin-flip and non spin-flip transition elements as


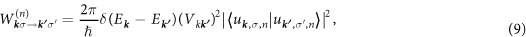



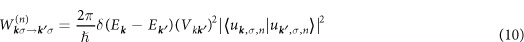


within the approximation obtained by applying the Fermi’s golden rule.

A rigorous derivation of the spin-relaxation rate 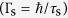 which characterizes the process of a not fully polarized spin ensemble of conduction electrons becoming in equilibrium with the environment due to interactions is obtained through a rate equation after ref. 3:


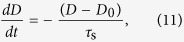


where 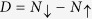 is the population difference between the conduction electrons with spin-down and spin-up states, *D*_0_ is the equilibrium value, and *τ*_s_ is the spin-relaxation time. Since it is a very hard task to determine the population change of the spin-down (spin-up) conduction electrons *dN*_↓(↑)_/*dt* in Eq. (11) under arbitrary value of SOC, i.e. keeping the actual change of the spin magnitude, we follow Elliott’s derivation of the spin-relaxation rate. Namely, we write the change of the population of the spin-down and spin-up conduction electrons as 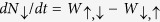 and 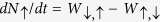, respectively, where *W*_↑,↓_(*W*_↑,↓_) is the number of transitions from spin-up (spin-down) state to spin-down (spin-up) state per unit time. Then, we express *W*_↑,↓_ and *W*_↑,↓_ by the spin-flip transition probabilities 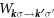 given in Eq. (9), i.e. we consider only the change of the Bloch states due to the SOC, and hence obtain the spin-flip rate by using Eq. (11). Elliott assumed that the spin-relaxation rate, Γ_s_, can be well approximated with the spin-flip rate, Γ_sf_, which reads:


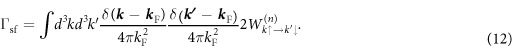


We note that in the limit of small SOC, the spin-flip rate Γ_sf_ given in Eq. (12) is the same as the spin-relaxation rate. Although it is not explicitly proven herein, we assume that Γ_s_ ≈ Γ_sf_, is retained even for a large SOC. We therefore refer to the definition of the spin-flip rate as spin-relaxation rate throughout in this paper.

Finally, the momentum-relaxation rate is obtained as


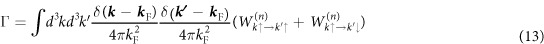


in Elliott’s picture.

## Results and Discussion

### The Elliott-Yafet model for strong SOC

We consider a four-band model which consists of two nearby bands each with a two-fold degeneracy with respect to the spin-up and spin-down states. The bands are separated by an energy gap Δ, and we neglect the dispersion of the bands. We found that this simplified model allows to derive analytic expression for the spin-relaxation rate for arbitrary value of the spin-orbit interaction. The most general form of the spin-orbit interaction with inversion symmetry is discussed in ref. 11:


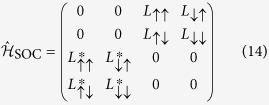


within the basis 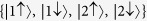 where 1 and 2 label the bands, and 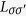 are SOC matrix elements.

For inversion and time-reversal symmetries, the SOC matrix elements satisfy: 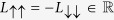 and 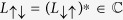. The detailed group theoretical considerations are given in the Supplementary Material. For simplicity, we keep only spin-flip SOC matrix elements which connect the states 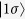 and 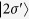 and neglect the ones which mix the same spin directions. We also consider a small Zeeman energy, *h*, to handle computational difficulties due to the doubly degenerate bands but we reinsert *h* = 0 at the end of the calculations.

**Figure 1:**
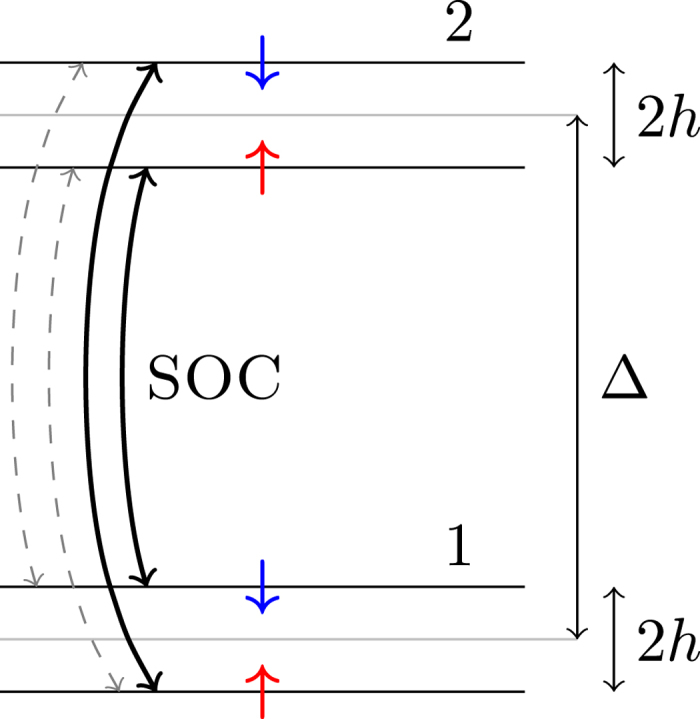
Schematics of the four-band model with SOC and a Zeeman term, *h*.

This Hamiltonian of this simplified model is then given by:


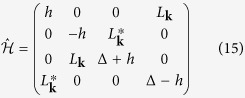


which includes kinetic, SOC, and the Zeeman terms. The level structure and the relevant terms of this model are depicted in Fig. 1.

We retain the ***k*** index for the SOC matrix elements in Eq. (15) to indicate that it depends on both the direction and magnitude of the wave vector, even though we consider dispersionless bands. This is required as otherwise 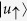 and 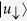 are orthogonal, which would give zero spin transition rate: 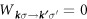.

The original energy splitting, Δ, is modified by the SOC as 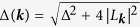. The Bloch functions in Eq. (7) are obtained as the eigenstates of Hamiltonian (15):


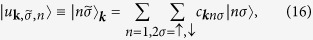


where the index 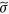 denotes mixed spin states due to the SOC. For example, the lowest two states are obtained as


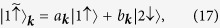



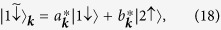


which have the same form as the ones given in Eqs (1) and (2) derived by Elliott. The explicit expressions for the coefficients *a*_***k***_ and *b*_***k***_ in Eqs (17) and (18) for arbitrary value of the SOC are given in the Supplementary Material. In the limit of small SOC, i.e. 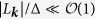, we recover the perturbation result of Elliott^2^ as 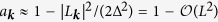 and 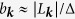.

The spin-flip and non spin-flip transition probabilities can be calculated at each band based on the formulae (9) and (10) and the general result is given in the Supplementary Material. Analytic results are obtained when i) the Fermi surface is approximated with a sphere with radius *k*_F_ which is a good approximation for (***k*** ≈ 0), ii) the unknown overlap integrals of the orbital part of the states and the impurity potential are estimated by constants as 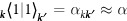, 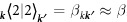, 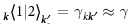, 
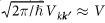
, and iii) the SOC matrix elements are approximated by their average values at the Fermi surface: 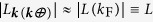. The latter two approximations are justified as these quantities are band structure dependent constants after the integration over the Fermi surface such as *α*_1_ is introduced in Eq. (3).

With these simplifications the transition probabilities read:


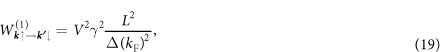



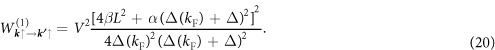


There is no spin relaxation 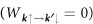 in the absence of SOC, i.e. *L* = 0.

Since the *k*-integrations in Eqs (12) and (13) cannot be performed in the simplified four-band model, we use the reasonable assumption that 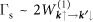 and 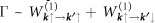 which will be justified by the subsequent numerical calculations. As a result, the ratio Γ_s_/Γ reads:


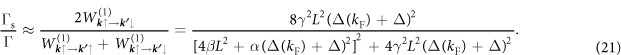


In the limit of small SOC, i.e 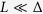, the ratio becomes:


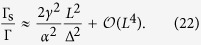


When neglecting the constants near unity, it leads to:


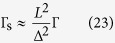


reproducing the perturbation result of Elliott given in Eq. (3). In this limit, the spin-relaxation rate is much smaller than the momentum-relaxation rate.

In the opposite limit, i.e. 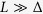, the ratio tends to a constant as a function of *L*:


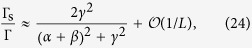


which means that the spin-relaxation rate approaches to the momentum-relaxation rate (apart from the constants *α*, *β*, and *γ*, which are near unity).

**Figure 2:**
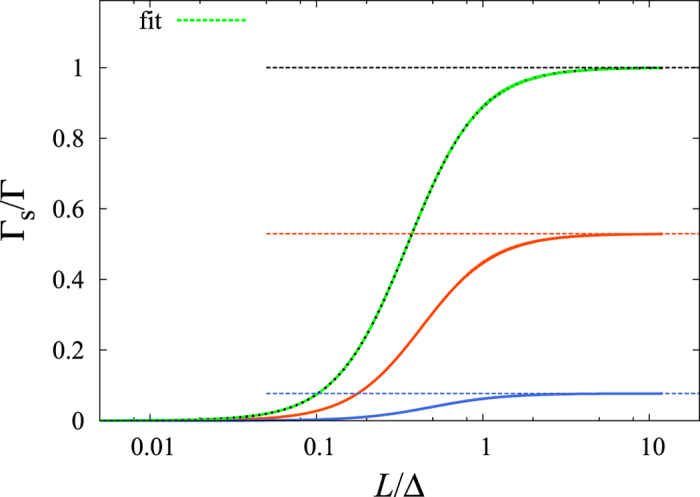
The ratio Γ_s_/Γ as a function of the SOC matrix element *L* with the parameter choice Δ = 1, *V* = 1, *α* = 0.5, *β* = 0.5, *γ* = 1 (*black*), *γ* = 0.6 (*red*), and *γ* = 0.2 (*blue*). *Horizontal dashed lines* show the large-*L* limits given by 2*γ*^2^/[(*α* + *β*)^2^ + *γ*^2^]. The perfect fit of the upper curve with the function in Eq. (25) is shown by *dashed green line*.

Figure 2 shows the ratio Γ_s_/Γ as a function of the SOC matrix element in the entire *L*-range with different values of the band-structure dependent constants. The characteristic energy where the behavior of the ratio changes from *L*^2^-dependence to saturation is approximately *L* ≈ Δ, i.e. where *L*/Δ ≈ 1. Figure 2 also shows a fit with the following phenomenological formula, which was found to well approximate the result in the entire parameter range:


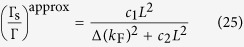


with *c*_1_ and *c*_2_ being constants of order of unity. We note that the simple phenomenological formula (25) is already implied by the analytic result given in Eq. (21) after some manipulations (see the Supplementary Material).

We also discuss the *g*-factor shift which is given by the expectation value of the orbital momentum in the Bloch state through the Zeeman term. A straigthforward calculation (detailed in the Supplementary Material) yields for 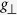 and 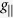, i.e. when the magnetic field is perpendicular or parallel to the *z* axis defined by the SOC Hamiltonian (Eq. (14)), respectively:


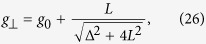



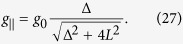


The EY theory predicts an isotropic *g*-factor and the anisotropy in our results is due to the omission of the *L*_↑↑_ and *L*_↓↓_ terms in Eq. (15). To test the Elliott-relation we proceed with *g*_⊥_ as:


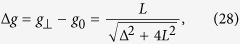


which becomes Δ*g* ~ *L*/Δ for 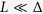 reproducing the Elliott result.

Combining expression (28) with (25) gives:


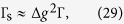


which recovers the Elliott relation given in Eq. (5) for arbitrary values of *L*.

### Pseudopotential electron model of spin relaxation

To complement the results of the model Hamiltonian calculations, we consider a nearly-free electron model where spin and momentum scattering can be calculated. The Hamiltonian of the problem is:


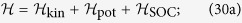



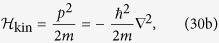



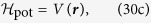



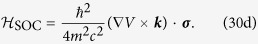


We solve it with the pseudopotential method which approximates the lattice potential *V*(***r***) with its first few Fourier components *V*(***g***) as


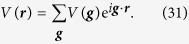


In the calculations we take 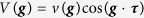 with 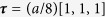, which corresponds to a zincblende structure with identical atoms (example Si or Ge), i.e. with inversion symmetry retained (for further details, see Supplementary Material).

This model has some advantages and shortcomings when calculating spin relaxation. An advantage is that the SOC is obtained directly from the lattice potential thus it allows to perform a numerical analysis of spin relaxation. In addition, the pseuodpotential-based SOC automatically accounts for the crystal symmetry which is found in the SOC, too. However, the pseudopotential-based SOC is known to underestimate the experimental SOC as it considers the smooth potential of the valence electrons only and neglects the strongly oscillating potential near the atomic core^12^. To solve this problem, the pseudopotential-based SOC is magnified with a scaling parameter with a typical value of *λ*_sc_ ~ 10^2^ − 10^3^. We chose *λ*_sc_ = 264 as it accounts quantitatively well for the *k* = 0 SOC gap in Ge^12^. In addition, we also introduce a *tuning parameter*, *λ*, for the SOC interaction in order to study the SOC dependent spin relaxation. As a result, the SOC Hamiltonian reads as 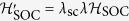 in Eq. (30d). We note that more realistic pseudopotentials were used successfully to reproduce the SOC strength in Si^13^.

**Figure 3:**
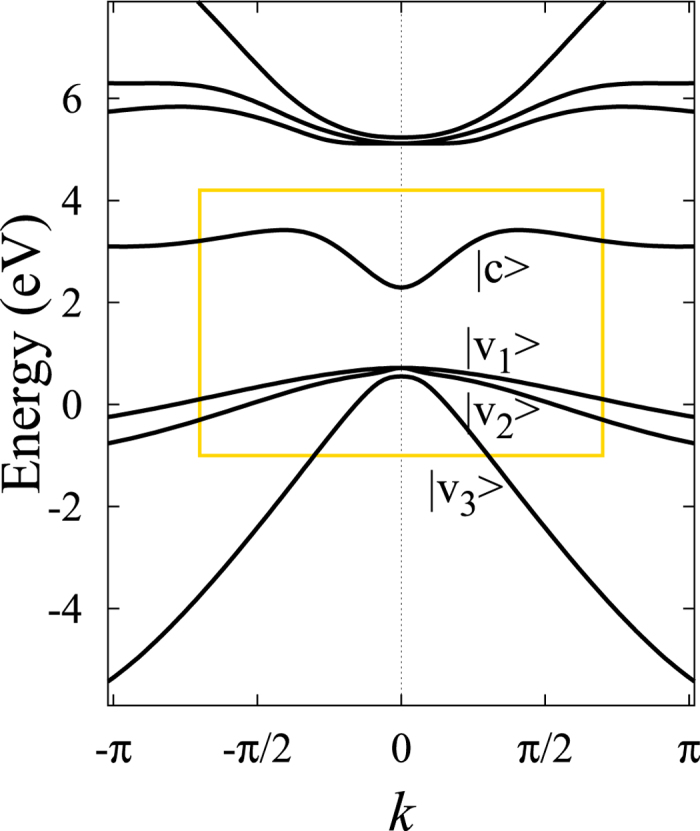
The band structure obtained with the used pseudopotential parameters for *λ* = 1 along the 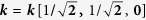 direction. |*v*_*n*_〉 and |*c*〉 denote the valence and conduction bands (each with additional spin degeneracy ↑, ↓), respectively.

We use a pseudopotential parameter set which reproduces well the conduction and valence bands of Ge. The band structure is shown in Fig. 3 for *λ* = 1. Considering the nearby valence and conduction bands denoted as 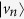 and |*c*〉 in Fig. 3, respectively, the characteristic features of metals with hybridized *s*-*p* orbitals can be discussed. Namely, the orbital parts of the electron wave functions at ***k*** = 0 (Γ point) without SOC have the symmetries |*c*〉 ~ Γ_1_ and |*v*_*n*_〉 ~ Γ_4_ which mean *s*- and *p*-like orbitals, respectively. Symmetry operations acting on the spin wave functions have to be considered in the presence of SOC, which can be handled by double group irreducible representations.

At ***k*** = 0, the *p*-symmetric (Γ_4_) valence band with spin splits into a four- and a two-fold degenerate state, separated by the SOC gap. These split states can be labeled according to the total angular momentum operator as *j* = 3/2 ~ Γ_8_ and *j* = 1/2 ~ Γ_7_, respectively. As we move away from the ***k*** = 0 point, the four-fold degenerate valence band splits further but a two-fold degeneracy is kept due to the presence of time-reversal symmetry. In the vicinity of the ***k*** = 0 point, all of the valence and conduction bands have mixed *s*-*p* character due to the hybridization.

Using Eqs (9) and (10), we derive the non spin-flip and spin-flip transition matrix elements in the conduction band |*c*〉 as


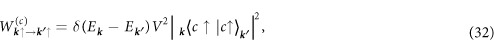



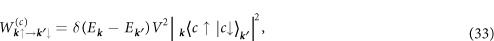


and in the upper valence band |*v*_1_〉 as


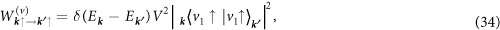



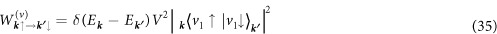


with 
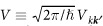
 that is not specified, but the ***k***-dependence of the wave functions is obtained accurately in the pseudopotential approximation.

The momentum- and spin-relaxation rates are obtained according to Eqs (12) and (13) by averaging over the transition matrix elements numerically with respect to ***k*** and ***k***′ on the Fermi surface with energy 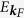 as:


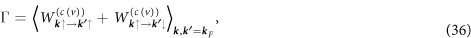



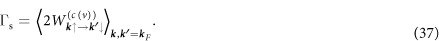


**Figure 4:**
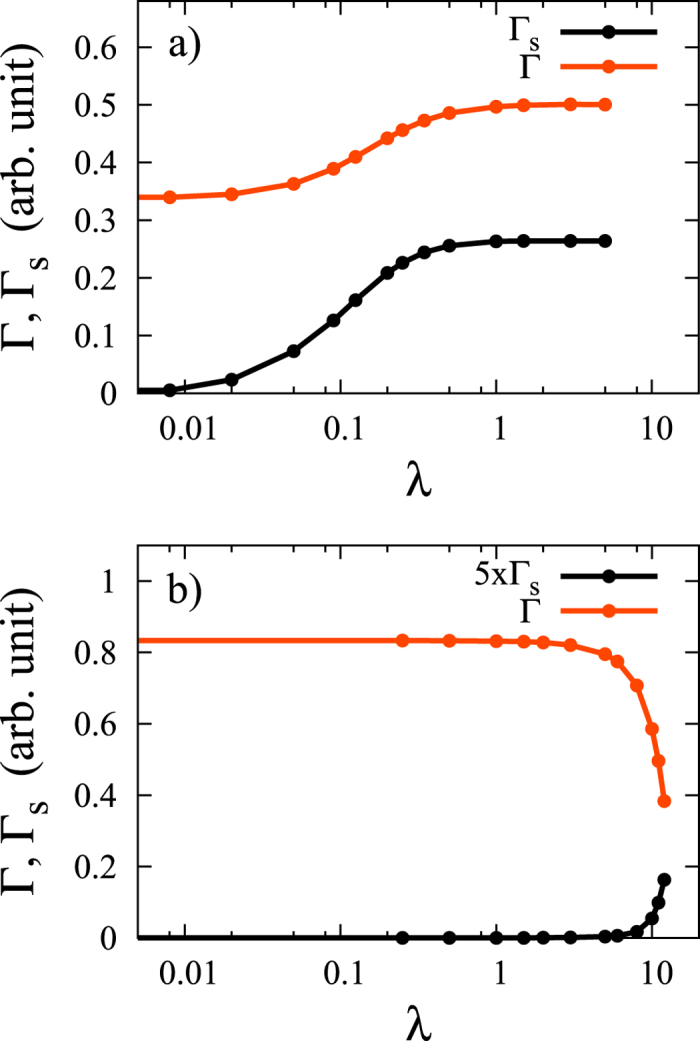
Spin- and momentum-relaxation rates in arbitrary units, calculated in the pseudopotential approximation as a function of spin-orbit coupling *λ* at *k*_F_ = 0.4 in the valence band (**a**) and in the conduction band (**b**).

Figure 4 shows the spin- and momentum-relaxation rates calculated in the pseudopotential approximation both for the upper valence band |*v*_1_〉 and conduction band |*c*〉 as a function of the spin-orbit coupling, *λ*. We took *k*_F_ = 0.4 in the calculation and averaged the transition probabilities given in Eqs (34) and (35) over 30 points at the Fermi surface to obtain the relaxation rates, Γ and Γ_s_.

The seemingly different behavior of the relaxation rates in the valence and conduction bands can be consistently explained. As it is shown in Fig. 3, the ‘relevant’ band gap, i.e. the characteristic energy defined as 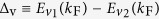 for the valence state and 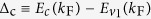 for the conduction band, is quite different for the two cases. Namely, in the case of the upper valence state, |*v*_1_〉, there is another valence state |*v*_2_〉 in its close vicinity, while the closest state to the conduction band |*c*〉 is |*v*_1_〉, which is much further away, which leads to 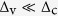. Thus, by increasing the SOC in the calculations, the range 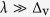 can be reached for the valence band and we indeed find the saturating behavior for both Γ and Γ_s_ in this range in well agreement with the result of the four-band model. On the other hand, in the case of the conduction band, the relation 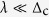 holds in the entire range of *λ* which we study. A much larger *λ* would lead to a rearranged band structure. Therefore, for the conduction band in our model, we observe the perturbative (or Elliott-Yafet) behavior 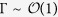 and 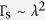.

**Figure 5:**
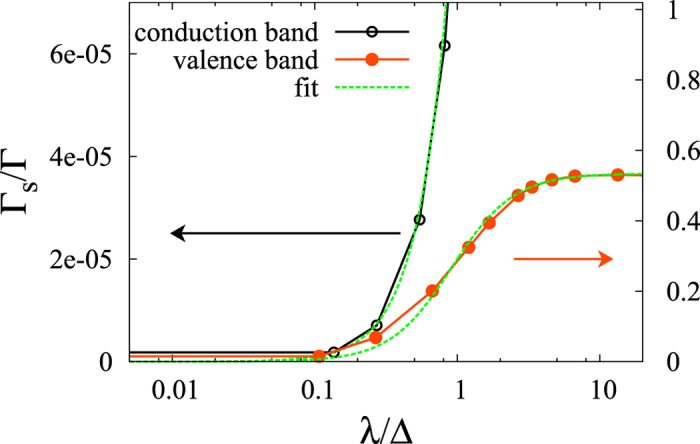
Fit to the ratio Γ_s_/Γ calculated in the pseudopotential approximation for the valence and conduction bands as a function of SOC strength (*λ*) at *k*_F_ = 0.4 with the formula (25).

We find that the numerical data for the ratio Γ_s_/Γ follows the approximate formula obtained in the model Hamiltonian calculation for both the valence and conductions bands. Namely, the approximate formula given in Eq. (25) fits well the ratios by taking the numerical data Δ_v_ and Δ_c_ for the band gap Δ(*k*_F_) for the valence and conduction band, respectively. Figure 5 shows the fit for the ratio Γ_s_/Γ as a function of *λ*/Δ with this model.

The good agreement between the numerically obtained data and the phenomenological formula (25) obtained in the four-band model calculation supports the previous assumption that 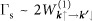 and 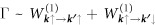. In addition, we find it compelling that the two different approaches result in Γ_s_/Γ ratios as a function of the SOC which are both well approximated by the formula in Eq. (25). This as we believe, summarizes the Elliott-Yafet theory generalized for the case of strong spin-orbit coupling.

### Comparison with experiment

Among all the metals where experimental data on spin relaxation exists, Au is particularly suited to test the validity of the above discussion as it has the strongest SOC. In addition, gold has a single conduction electron per unit cell, i.e. its Fermi surface does not extend beyond the first Brillouin zone. It is known that description of spin relaxation is more complicated for metals with two or more conduction electrons per unit cell^14^,^15^ (e.g. Mg or Al), where the Fermi surface crosses the Brillouin zone boundaries giving rise to the so-called spin relaxation “hot-spots”. When hot-spots are present, the Elliott relation is known to break down even though the Elliott-Yafet theory remains valid^14^,^15^.

Monod and Beuneu found^9^ the SOC strength in Au as *L*/Δ ≈ 0.9. It is therefore an appropriate candidate for a case where the perturbative treatment of the SOC breaks down. Monod and Jánossy reported in ref. 10 the electron spin resonance linewidth, Δ*B*, which is used to obtain Γ_s_. Γ is calculated from the resistivity, *ρ*, data in ref. 16 through: 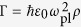, where *ε*_0_ is the vacuum permittivity and *ω*_p1_ = 8.55 eV is the plasma frequency of gold^17^. For both sets of data, the residual linewidth and resistivity was subtracted as both quantities are known to obey the Matthiessen’s rule^7^, i.e. that the respective residual and temperature dependent scattering rates are additive.

**Figure 6:**
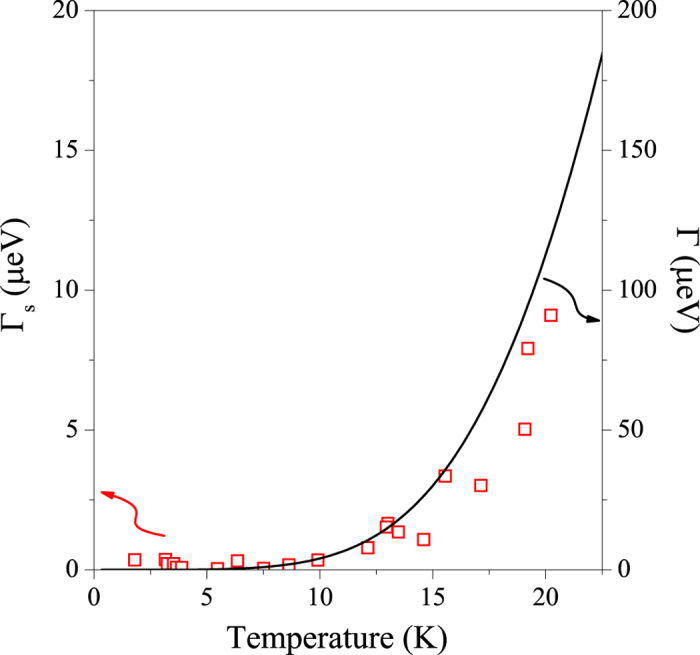
Experimentally determined spin-lattice relaxation rate, Γ_s_, in Au from ref. 10 as compared to the momentum scattering rate, Γ. Note the factor 10 difference in the energy scales for the two quantities.

The comparison, shown in Fig. 6, shows that the measured spin-relaxation rate approaches the momentum-relaxation rate within an order of magnitude such as our theoretical result suggests. The difference between the calculated Γ_s_/Γ ≈ 1 and the observed Γ_s_/Γ = 0.06 ± 0.01 could be due to the band structure dependent constants near unity in Eq. (24). However the observation of a nearly equal Γ_s_ and Γ is itself surprising as to our knowledge it has not been noted elsewhere that the mergence of spin and momentum relaxation times could be realized for a real material.

We finally test empirically the validity of the Elliott relation in Eq. (29), i.e. 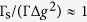 in gold. Monod and Jánossy found *g* = 2.11 ± 0.01 which gives 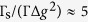, which is again in agreement with the theoretical prediction given the presence of the band structure dependent constant factors. This means that the Elliott relation remains a valid empirical tool to test whether the Elliott-Yafet theory applies for a given system.

## Conclusions

We generalized the Elliott-Yafet theory of spin relaxation in metals with inversion symmetry for the case of arbitrary value of the spin-orbit coupling. We applied two different approaches, exact diagonalization of a model four-band Hamiltonian (without dispersion) and numerical calculation of the spin relaxation time for a pseudopotential approach. The two methods give a qualitatively similar result, which is summarized in Eq. (25). A calculation of the *g*-factor in the four-band model shows that the empirical Elliott-relation, which links the *g*-factor and spin-relaxation rate, is retained even for strong SOC. Our result indicates that spin and momentum relaxation times can have similar orders of magnitude. We show that this situation has been already observed in Au. Our result is an important step toward the unified theory of spin relaxation including the strength of the spin-orbit coupling.

## Additional Information

**How to cite this article**: Kiss, A. *et al*. The Elliott-Yafet theory of spin relaxation generalized for large spin-orbit coupling. *Sci. Rep*. **6**, 22706; doi: 10.1038/srep22706 (2016).

## Supplementary Material

Supplementary Information

## References

[b1] Feher G. & Kip A. F. Electron Spin Resonance Absorption in Metals. I. Experimental. Phys. Rev. 98, 337–348 (1955).

[b2] Elliott R. J. Theory of the Effect of Spin-Orbit Coupling on Magnetic Resonance in Some Semiconductors. Phys. Rev. 96, 266–279 (1954).

[b3] Yafet Y. g-factors and spin-lattice relaxation of conduction electrons. Sol. St. Phys. 14, 1–98 (1963).

[b4] Žutić I., Fabian J. & Das Sarma S. Spintronics: Fundamentals and applications. Rev. Mod. Phys. 76, 323–410 (2004).

[b5] Wu M. W., Jiang J. H. & Weng M. Q. Spin dynamics in semiconductors. Phys. Rep. 493, 61–236 (2010).

[b6] Dyakonov M. & Perel V. Spin relaxation of conduction electrons in noncentrosymmetric semiconductors. Sov. Phys. Sol. St. (USSR) 13, 3023–3026 (1972).

[b7] Simon F. . Generalized Elliott-Yafet Theory of Electron Spin Relaxation in Metals: Origin of the Anomalous Electron Spin Lifetime in MgB_2_. Phys. Rev. Lett. 101, 177003 (2008). 18999776 10.1103/PhysRevLett.101.177003

[b8] Dóra B. & Simon F. Electron-Spin Dynamics in Strongly Correlated Metals. Phys. Rev. Lett. 102, 137001 (2009). 19392394 10.1103/PhysRevLett.102.137001

[b9] Monod P. & Beuneu F. Conduction-electron spin flip by phonons in metals: Analysis of experimental data. Phys. Rev. B 19, 911–916 (1979).

[b10] Monod P. & Janossy A. Conduction electron spin resonance in gold. J. Low Temp. Phys. 26, 311 (1977).

[b11] Boross P., Dóra B., Kiss A. & Simon F. A unified theory of spin-relaxation due to spin-orbit coupling in metals and semiconductors. Sci. Rep. 3, 3233 (2013). 24252975 10.1038/srep03233 PMC3834866

[b12] Yu P. Y. & Cardona M. Fundamentals of Semiconductors, Physics and Material Properties (Springer-Verlag: Berlin Heidelberg, , 2010).

[b13] Cheng J. L., Wu M. W. & Fabian J. Theory of the spin relaxation of conduction electrons in silicon. Phys. Rev. Lett. 104, 016601 (2010). 20366376 10.1103/PhysRevLett.104.016601

[b14] Fabian J. & Das Sarma S. Spin relaxation of conduction electrons in polyvalent metals: Theory and a realistic calculation. Phys. Rev. Lett. 81, 5624–5627 (1998).

[b15] Fabian J. & Das Sarma S. Phonon-induced spin relaxation of conduction electrons in aluminum. Phys. Rev. Lett. 83, 1211–1214 (1999).

[b16] Matula R. A. Electrical resistivity of copper, gold, palladium, and silver. J. Phys. Chem. Ref. Data 8, 1147–1298 (1979).

[b17] Blaber M. G., Arnold M. D. & Ford M. J. Search for the ideal plasmonic nanoshell: The effects of surface scattering and alternatives to gold and silver. J. Phys. Chem. C 113, 3041–3045 (2009).

