# Supplementary material for: The Elliott-Yafet theory of spin relaxation generalized for large spin-orbit coupling
Source: Sci Rep. 2016 Mar 4;6:22706. doi: 10.1038/srep22706 (PMC4778627; doi:10.1038/srep22706)
Supplement: Supplementary Information [file srep22706-s1.pdf]

Supplementary material for: The Elliott-Yafet theory of spin relaxation generalized for large spin-orbit coupling

Annamária Kiss,[1,2] Lénard Szolnoki,[3] and Ferenc Simon[3,*]

[1]*Institute for Solid State Physics and Optics, Wigner Research Centre for Physics, Hungarian Academy of Sciences, POBox 49, H-1525 Budapest, Hungary*

[2]*BME-MTA Exotic Quantum Phases Research Group, Budapest University of Technology and Economics, Budapest, Hungary*

[3]*Department of Physics, Budapest University of Technology and Economics and MTA-BME Lendület Spintronics Research Group (PROSPIN), POBox 91, H-1521 Budapest, Hungary*

I. GROUP THEORETICAL CONSIDERATIONS ON THE SOC MATRIX ELEMENTS

The Hamiltonian of the spin-orbit interaction given in Eq. (14) in the main paper is restricted by the following three properties: i) it is self adjoint, ii) it can be written in the form of $H_1 \otimes \sigma_x + H_2 \otimes \sigma_y + H_3 \otimes \sigma_z$ (for every im H_i is a kinetic operator (2×2) and $\otimes$ denotes tensorial product), and iii) it is invariant to the product of inversion and time-reversal operations.

The effect of inversion (R_i) and time-reversal (R_τ) operations on our basis are given by:

$$\begin{aligned} R_i R_\tau \left|1\uparrow\right\rangle &= \left\langle 1\downarrow\right|, \\ R_i R_\tau \left|1\downarrow\right\rangle &= -\left\langle 1\uparrow\right|, \\ R_i R_\tau \left|2\uparrow\right\rangle &= -\left\langle 2\downarrow\right|, \\ R_i R_\tau \left|2\downarrow\right\rangle &= \left\langle 2\uparrow\right|, \end{aligned} \tag{1}$$

where $R_i R_\tau$ denotes the product of inversion and time reversal.

Note that time reversal allows arbitrary $e^{i\varphi_{1,2}}$ factors for the two kinetic basis states, $|1\rangle$ and $|2\rangle$, depending how the basis were chosen. Using the notation used in main text, the second property yields $L_{\uparrow\uparrow} = -L_{\downarrow\downarrow}$. The third property implies:

$$\begin{aligned} \left\langle 1\uparrow\right| \hat{\mathcal{H}}_{\mathrm{SOC}} \left|2\uparrow\right\rangle &= -\left\langle 2\downarrow\right| \hat{\mathcal{H}}_{\mathrm{SOC}} \left|1\downarrow\right\rangle, \\ \left\langle 1\uparrow\right| \hat{\mathcal{H}}_{\mathrm{SOC}} \left|2\downarrow\right\rangle &= \left\langle 2\uparrow\right| \hat{\mathcal{H}}_{\mathrm{SOC}} \left|1\downarrow\right\rangle, \end{aligned} \tag{2}$$

which in our notation for the matrix elements reads:

$$\begin{aligned} L_{\uparrow\uparrow} &= -L^*_{\downarrow\downarrow}, \\ L_{\downarrow\uparrow} &= L^*_{\uparrow\downarrow}. \end{aligned} \tag{3}$$

This, together with the second restriction yields the result quoted in the main text:

$$\begin{aligned} L_{\uparrow\uparrow} &= -L_{\downarrow\downarrow} \quad &\in \mathbb{R}, \\ L_{\downarrow\uparrow} &= L^*_{\uparrow\downarrow} \quad &\in \mathbb{C}. \end{aligned} \tag{4}$$

II. DETAILS OF THE CALCULATIONS IN THE FOUR-BAND MODEL

The coefficients of the mixed spin states in Eqs. (17) and (18) of the main paper are obtained as

$$a_{\boldsymbol{k}} = -\frac{1}{\sqrt{2}} \left(1 + \frac{\Delta}{\sqrt{\Delta^2 + 4|L_{\boldsymbol{k}}|^2}}\right)^{1/2} = -\frac{1}{\sqrt{2}} \left(1 + \frac{\Delta}{\Delta(\boldsymbol{k})}\right)^{1/2}, \tag{5}$$

$$b_{\boldsymbol{k}} = \frac{1}{\sqrt{2}} \frac{L^*_{\boldsymbol{k}}}{|L_{\boldsymbol{k}}|} \left(1 - \frac{\Delta}{\sqrt{\Delta^2 + 4|L_{\boldsymbol{k}}|^2}}\right)^{1/2} = \frac{1}{\sqrt{2}} \frac{L^*_{\boldsymbol{k}}}{|L_{\boldsymbol{k}}|} \left(1 - \frac{\Delta}{\Delta(\boldsymbol{k})}\right)^{1/2}. \tag{6}$$

The spin-flip and non spin-flip transition elements in the lowest band calculated from the eigenstates read:

$$W^{(1)}_{k\uparrow\to k'\downarrow} = \frac{2\pi}{\hbar}\delta(E_{\boldsymbol{k}}-E_{\boldsymbol{k}'})\left(V_{\boldsymbol{kk}'}\right)^2 |_{\boldsymbol{k}}\langle 1\widetilde{\uparrow}|1\widetilde{\downarrow}\rangle_{\boldsymbol{k}'}|^2 = \frac{2\pi}{\hbar}\delta(E_{\boldsymbol{k}}-E_{\boldsymbol{k}'})\left(V_{\boldsymbol{kk}'}\right)^2 |a^*_{\boldsymbol{k}}b^*_{\boldsymbol{k}'}\,{}_{\boldsymbol{k}}\langle 1|2\rangle_{\boldsymbol{k}'} + b^*_{\boldsymbol{k}}a^*_{\boldsymbol{k}'}\,{}_{\boldsymbol{k}}\langle 2|1\rangle_{\boldsymbol{k}'}|^2$$
$$= \frac{2\pi}{\hbar}\delta(E_{\boldsymbol{k}}-E_{\boldsymbol{k}'})\left(V_{\boldsymbol{kk}'}\right)^2 \frac{|\gamma_{\boldsymbol{kk}'}L_{\boldsymbol{k}'}\left(\Delta(\boldsymbol{k})+\Delta\right)+\gamma^*_{\boldsymbol{k}'\boldsymbol{k}}L_{\boldsymbol{k}}\left(\Delta(\boldsymbol{k}')+\Delta\right)|^2}{\Delta(\boldsymbol{k})\Delta(\boldsymbol{k}')\left(\Delta(\boldsymbol{k})+\Delta\right)\left(\Delta(\boldsymbol{k}')+\Delta\right)}, \tag{7}$$

$$W^{(1)}_{k\uparrow\to k'\uparrow} = \frac{2\pi}{\hbar}\delta(E_{\boldsymbol{k}}-E_{\boldsymbol{k}'})\left(V_{\boldsymbol{kk}'}\right)^2 |_{\boldsymbol{k}}\langle 1\widetilde{\uparrow}|1\widetilde{\uparrow}\rangle_{\boldsymbol{k}'}|^2 = \frac{2\pi}{\hbar}\delta(E_{\boldsymbol{k}}-E_{\boldsymbol{k}'})\left(V_{\boldsymbol{kk}'}\right)^2 |a^*_{\boldsymbol{k}}a_{\boldsymbol{k}'}\,{}_{\boldsymbol{k}}\langle 1|1\rangle_{\boldsymbol{k}'} + b^*_{\boldsymbol{k}}b_{\boldsymbol{k}'}\,{}_{\boldsymbol{k}}\langle 2|2\rangle_{\boldsymbol{k}'}|^2$$
$$= \frac{2\pi}{\hbar}\delta(E_{\boldsymbol{k}}-E_{\boldsymbol{k}'})\left(V_{\boldsymbol{kk}'}\right)^2 \frac{|4\beta_{\boldsymbol{kk}'}L_{\boldsymbol{k}}L^*_{\boldsymbol{k}'}+\alpha_{\boldsymbol{kk}'}\left(\Delta(\boldsymbol{k})+\Delta\right)\left(\Delta(\boldsymbol{k}')+\Delta\right)|^2}{4\Delta(\boldsymbol{k})\Delta(\boldsymbol{k}')\left(\Delta(\boldsymbol{k})+\Delta\right)\left(\Delta(\boldsymbol{k}')+\Delta\right)}, \tag{8}$$

where we introduced the notations ${}_{\boldsymbol{k}}\langle 1|1\rangle_{\boldsymbol{k}'} \equiv \alpha_{\boldsymbol{kk}'}$, ${}_{\boldsymbol{k}}\langle 2|2\rangle_{\boldsymbol{k}'} \equiv \beta_{\boldsymbol{kk}'}$, and ${}_{\boldsymbol{k}}\langle 1|2\rangle_{\boldsymbol{k}'} \equiv \gamma_{\boldsymbol{kk}'}$ for the unknown overlap of states with different wave vectors $\boldsymbol{k}, \boldsymbol{k}'$.

With the assumptions detailed in the main text, we obtain for the ratio of spin- and momentum-relaxation rates as:

$$\frac{\Gamma_{\rm s}}{\Gamma} \approx \frac{2W^{(1)}_{\boldsymbol{k}\uparrow\to\boldsymbol{k}'\downarrow}}{W^{(1)}_{\boldsymbol{k}\uparrow\to\boldsymbol{k}'\uparrow}+W^{(1)}_{\boldsymbol{k}\uparrow\to\boldsymbol{k}'\downarrow}} = \frac{8\gamma^2L^2\left(\Delta(k_{\rm F})+\Delta\right)^2}{\left[4\beta L^2+\alpha\left(\Delta(k_{\rm F})+\Delta\right)^2\right]^2+4\gamma^2L^2\left(\Delta(k_{\rm F})+\Delta\right)^2}. \tag{9}$$

This can be rewritten as:

$$\frac{\Gamma_{\rm s}}{\Gamma} = \frac{8\gamma^2L^2}{\left[\alpha^2\left(\Delta(k_{\rm F})+\Delta\right)^2+4(2\alpha\beta+\gamma^2)L^2+\frac{(4\beta)^2L^4}{(\Delta(k_{\rm F})+\Delta)^2}\right]}. \tag{10}$$

The last term in the denominator of Eq. (10) is proportional to L^4 for $L \ll \Delta$ in which limit it can be neglected because the leading SOC term is L^2, and proportional to L^2 for $L \gg \Delta$ where it can be summed up together with the original L^2 term. This justifies the form of the empirical fitting function for $\Gamma_{\rm s}/\Gamma$ in the main text.

The g-factor is obtained from the Zeeman Hamiltonian containing both the spin and orbital term with the respective operators (S and L):

$$\begin{aligned}\mathcal{H}_{\rm Z} &= -\mu_{\rm B}\boldsymbol{B}\cdot(\boldsymbol{L}+g_0\boldsymbol{S}) \\ &= -\mu_{\rm B}\left(\frac{1}{2}\left[B_+(L_++g_0S_+)+B_-(L_-+g_0S_-)\right]\right. \\ &\quad \left.+\ B_z(L_z+g_0S_z)\right).\end{aligned} \tag{11}$$

The spin-flip SOC matrix element in our four-band model connects the states $|1\sigma\rangle$ and $|2\sigma'\rangle$, which means that only the orbital momentum operators L_+ and L_- are to be considered in Eq. (11).

Let us change the direction of the magnetic field in the $x-z$ plane from the z axis, which gives $H_z = H\cos\theta$, and $H_x = H\sin\theta$. The expectation values of the Zeeman term between the states $|1\widetilde{\uparrow}\rangle$ and $|1\widetilde{\downarrow}\rangle$ given in Eqs. (17), (18) in the main paper are calculated as

$$\begin{aligned}\langle 1\widetilde{\sigma}|\mathcal{H}_{\rm Z}|1\widetilde{\sigma}\rangle &= -\langle 1\widetilde{\sigma}'|\mathcal{H}_{\rm Z}|1\widetilde{\sigma}'\rangle \\ &= -\mu_{\rm B}g_0H\cos\theta(a^2_{\boldsymbol{k}}-b^2_{\boldsymbol{k}}),\end{aligned} \tag{12}$$
$$\langle 1\widetilde{\sigma}|\mathcal{H}_{\rm Z}|1\widetilde{\sigma}'\rangle = -\mu_{\rm B}H\sin\theta\left[g_0(a^2_{\boldsymbol{k}}+b^2_{\boldsymbol{k}})-a_{\boldsymbol{k}}b_{\boldsymbol{k}}\right]. \tag{13}$$

Diagonalizing this 2×2 problem, we obtain the modified Zeeman energies as

$$\varepsilon_{\rm Z} = \pm\mu_{\rm B}H\sqrt{g^2_\perp\sin^2\theta+g^2_\parallel\cos^2\theta} \tag{14}$$

with

$$g_\perp = g_0+\frac{L}{\sqrt{\Delta^2+4L^2}}, \tag{15}$$
$$g_\parallel = g_0\frac{\Delta}{\sqrt{\Delta^2+4L^2}}. \tag{16}$$

III. DETAILS OF THE CALCULATIONS IN THE PSEUDOPOTENTIAL ELECTRON MODEL

We take the first five reciprocal vectors $\boldsymbol{g}$: $[\boldsymbol{g}_0(1), \{\boldsymbol{g}_3(8)\}, \{\boldsymbol{g}_4(6)\}, \{\boldsymbol{g}_8(12)\}, \{\boldsymbol{g}_{11}(24)\}]$, where the degeneracy of each reciprocal vector is indicated, in the Fourier expansion of the lattice potential, which leads to 102 lowest-lying states (51×2, where 2 corresponds to the spin degrees of freedom).

Taking the electron wave functions given in Eq. (7) of the main paper, the Schrödinger equation simplifies for each $\boldsymbol{k}$ to the eigenvalue problem of the Hamiltonian matrix

$$\begin{aligned}\mathcal{H}_{\boldsymbol{g}\sigma,\boldsymbol{g}'\sigma'}(\boldsymbol{k}) &= \left(\mathcal{H}_{\rm kin}\right)_{\boldsymbol{g}\sigma,\boldsymbol{g}'\sigma'}(\boldsymbol{k})+\left(\mathcal{H}_{\rm pot}\right)_{\boldsymbol{g}\sigma,\boldsymbol{g}'\sigma'}(\boldsymbol{k}) \\ &\quad+\left(\mathcal{H}_{\rm SOC}\right)_{\boldsymbol{g}\sigma,\boldsymbol{g}'\sigma'}(\boldsymbol{k})\end{aligned} \tag{17}$$

obtained from Eq. (30a) of the main paper, where

$$\left(\mathcal{H}_{\rm kin}\right)_{\boldsymbol{g}\sigma,\boldsymbol{g}'\sigma'}(\boldsymbol{k}) = \frac{\hbar^2}{2m}(\boldsymbol{k}+\boldsymbol{g})^2\delta_{\boldsymbol{g},\boldsymbol{g}'}\delta_{\sigma,\sigma'} \tag{18}$$
$$\left(\mathcal{H}_{\rm pot}\right)_{\boldsymbol{g}\sigma,\boldsymbol{g}'\sigma'}(\boldsymbol{k}) = V(\boldsymbol{g}-\boldsymbol{g}')\delta_{\sigma,\sigma'} \tag{19}$$
$$\begin{aligned}\left(\mathcal{H}_{\rm SOC}\right)_{\boldsymbol{g}\sigma,\boldsymbol{g}'\sigma'}(\boldsymbol{k}) &= \lambda_{\rm sc}\lambda\frac{\hbar^2}{4m^2c^2}\left[(\boldsymbol{k}+\boldsymbol{g})\times(\boldsymbol{k}+\boldsymbol{g}')\right] \\ &\quad\cdot\ \boldsymbol{\sigma}_{\sigma,\sigma'}\left[-iV(\boldsymbol{g}-\boldsymbol{g}')\right].\end{aligned} \tag{20}$$

The eigenstates and eigenvalues are obtained by numerical diagonalization of the 102×102 matrix $\mathcal{H}_{\boldsymbol{g}\sigma,\boldsymbol{g}'\sigma'}(\boldsymbol{k})$.
